# Pathogenic mucorales: Deciphering their cell wall polysaccharidome and immunostimulatory potential

**DOI:** 10.1080/21505594.2025.2528079

**Published:** 2025-07-07

**Authors:** Mathieu Lepas, Julia Marcondes Figueiredo, Sarah Dellière, Sarah Sze Wah Wong, Dea Garcia-Hermoso, Magalie Duchateau, Mariette Matondo, Fanny Lanternier, Vishukumar Aimanianda

**Affiliations:** a Institut Pasteur, Université Paris Cité, Molecular Mycology Unit, UMR CNRS2000, Paris, France; b Institut Pasteur, Université Paris Cité, Immunobiology of Aspergillus, Paris, France; c Department of Microbiology, Institute of Biomedical Sciences, University of São Paulo, São Paulo, Brazil; d Mycology Department, Institut Pasteur, Université Paris Cité, National Reference Center for Invasive Mycoses and Antifungals, Translational Mycology research group, Paris, France; e Institut Pasteur, Proteomics Platform, Mass Spectrometry for Biology Unit, CNRS URA 2024, Paris, France; f AP-HP, Service de maladies infectieuses et tropicales, Hopital Necker, Paris, France; g Department of Biochemistry, Kasturba Medical College Manipal, Manipal Academy of Higher Education, Manipal, India

**Keywords:** Mucorales, mucormycosis, cell wall, polysaccharides, β-glucan, immunomodulation

## Abstract

Mucormycosis is an emerging infection caused by pathogenic filamentous fungal species belonging to the Order Mucorales. Mortality associated with mucormycosis is significantly high in patients with compromised immunity. As cell wall is the first fungal component to interact with the host immune system, we characterized cell wall organization and composition of the three most prevalent pathogenic species of Mucorales, *Rhizopus arrhizus*, *Mucor circinelloides, and Lichtheimia corymbifera* and studied their immunomodulatory potential. Staining and lectin-/immunolabeling indicated that the spores and germ-tubes of these three Mucorales species have surface-exposed mannans, while germ-tubes showed distinctly distributed β-1,3-glucan. Gas chromatography analysis of the cell wall indicated that glucose polymer is the major fibrillar polysaccharide present in the three species, whereas amorphous components were species-dependent. Specific enzymatic digestion followed by chromatography analysis indicated that β-1,3-glucan, β-1,6-glucan, and amylase digestible glucan constitute firbrillar polysaccharides. Stimulation of human peripheral blood mononuclear cells or THP-1 (human leukemic monocytic) cells with spores or extracted cell wall polysaccharides resulted in the release of pro-inflammatory cytokines regardless of the Mucorales species. Together, the Mucorales species analyzed in this study show a common and species-specific cell wall composition. The cell wall polysaccharides are highly pro-inflammatory, suggesting that undue or excessive inflammation may contribute to the immunopathology of mucormycosis.

## Introduction

Mucorales are mucormycotic filamentous fungal order comprising about 261 species under 55 genera. Of them, ~38 species have been reported to cause infections in humans [1]. Mucormycosis is a life-threatening disease, the presentation of which varies from invasive cutaneous infection (occurring following trauma) to rhino-orbital cerebral, pulmonary, gastrointestinal, or disseminated mucormycosis leading to angioinvasion, vessel thrombosis, and tissue necrosis [2–4]. This fungal infection is associated with unacceptably high mortality rate [5]. The most prevalent pathogenic species of Mucorales belong to the genera *Rhizopus*, *Mucor*, *Lichtheimia,* and *Rhizomucor* [2,6–8]. The susceptible hosts are those with immune defects as in hematologic malignancies, diabetes mellitus, corticosteroid therapy, or transplant patients [5,9,10]. COVID-19 associated mucormycosis (CAM) has been reported [11]. The prognosis of mucormycosis remains poor due to diagnostic issues delaying treatment, limited options of antifungal drug, tissue necrosis limiting the drug efficacy, and poor recovery from underlying diseases. Surgical debridement of infected tissues increases outcome, but compromises life quality. Therefore, there is a need to study the pathobiology of Mucorales for better management of mucormycosis.

Skin and mucosal surfaces are the physical barriers protecting humans from any microbial pathogens. Damage or disruption of these barriers leads to microbial breaching, during when host innate immune system offers a protective function. On the other hand, fungi are endowed with a cell wall, the composition and organization of which are species specific [12]. This cell wall protects fungus from host recognition or immunological insults. For example, (i) the rodlet layer present on the surface of *Aspergillus fumigatus* conidia masks them from immediate host immune recognition [13]; (ii) the capsular structure anchored to the cell wall of *Cryptococcus neoformans* avoids this fungal recognition and uptake by host phagocytes [14]; (iii) when attacked by neutrophils, *Candida albicans* unmasks the cell wall β-1,3-glucan, a pathogen-associated molecular pattern (PAMP); however, there will be compensatory chitin synthesis, resulting in cell wall remodeling in response to host immune attack [15]; (iv) during infection, α-1,3-glucan gets deposited on the *Histoplasma capsulatum* yeast surfaces, which blocks cell wall β-1,3-glucan from immune recognition [16].

Above studies suggest a crucial role played by the fungal cell wall during host-fungal interaction. With regard to Mucorales, studies performed were focused only on specific cell wall components [17]. For example, *Rhizopus arrhizus* stimulates IL-23 secretion by human dendritic cells (DCs) and drives Th17 response upon selectively exposing its cell wall β-glucan that activates dectin-1 exposed on DCs [18]. However, the cell wall of Mucorales is composed not only of β-1,3-glucan, but with different polysaccharides [17]. On the other hand, pathogenic Mucorales have shown to be resistant to a wide range of antifungal drugs, while antifungal drugs target fungal cell walls directly or indirectly. For example, lipid formulations of amphotericin B and triazoles (posaconazole and isavuconazole) used to treat mucormycosis destabilize the cell wall organization [19]. A global review reported that the species of *Rhizopus*, *Mucor,* and *Litchtheimia* account for 75% of the mucormycosis [20]. However, their clinical presentation varies; *Rhizopus* and *Mucor* species are the principal pathogens causing rhino-orbital cerebral mucormycosis [21] and the most common etiological agent of invasive mucormycosis is the species of *Rhizopus*, followed by *Mucor* and *Lichtheimia* species [22,23]. The species from all these three genera of Mucorales have been reported to cause cutaneous mucormycosis [24,25]. We aimed at fulfilling the research gap of exploring the role played by the cell walls of Mucorales species during host-fungal interaction, focusing on three frequent pathogenic species of Mucorales, *R. arrhizus*, *M. circinelloides,* and *L. corymbifera*.

## Materials and methods

Isolates: The clinical isolates of Mucorales species studied are presented in Table 1. These clinical isolates were obtained from the Centre national de référence des Mycoses invasives et antifongiques (CNRMA; The National Reference Centre for Invasive Mycoses and Antifungals) affiliated to Institut Pasteur Paris, with the consent from the medical mycologist who sent them to the CNRMA. They were subcultured on 2% Malt Extract agar (MEA) slants and incubated at 30°C for 4–5 days. Spores were collected from these MEA slants using aqueous Tween-80 (0.05%). Spore suspensions were passed through a 40-μm cell strainer (ThermoFisher Scientific) to remove any mycelia, washed with aqueous Tween-80 (0.05%), counted using automated cell counter (Luna-FL), and used immediately for further studies.

**Table 1.** Minimum inhibitory/effective concentration (MIC/MEC50) values of different antifungal drugs against clinical isolates used in this study.

			AmB	Itra	Vori	Posa	Isavu	Cas	Mica	Terb
Species	Isolate	Isolation site		(mg/L)						
*Rhizopus arrhizus*	CNRMA20.506	Lung	0.25	4	16	1	4	8	8	16
*Mucor circinelloides*	CNRMA20.753	BAL	0.125	16	16	16	8	16	16	16
*Lichtheimia corymbifera*	CNRMA19.751	Lung	0.25	1	16	0.5	4	8	8	0.25

BAL-Bronchoalveolar lavage fluid; Amphotericin-B-AmB; Itraconazole-Itra; Voriconazole-Vori; Posaconazole-Posa; Isavuconazole-Isavu; Caspofungin-Cas; Micafungin-Mica; Terbenafine-Terb.

### Determination of minimum inhibitory or effective concentrations (MIC or MEC, respectively)

The assay was performed according to the European Committee on Antimicrobial Susceptibility Testing (EUCAST) with modifications [26]. After 24–48 h of incubation at 35°C, the absorbance was measured at 405 nm. Minimum inhibitory concentrations (MICs) were recorded for polyene (amphotericin B), azoles, and terbinafine, and minimum effective concentrations (MECs) were recorded for echinocandins (caspofungin and micafungin). All antifungal drugs were purchased from Alsachim (Illkirch-Graffenstaden, France).

### Staining/Labeling and microscopic analyses

Bright-field microscopy was performed for Mucorales spores. Spores sizes were determined using ImageJ software by counting at least 100 spores for each isolate. For germ-tube preparation, 1x10^5^ spores/mL suspended in RPMI 1640-MOPS-2% glucose were seeded in a well of *μ*-slide 8-well plates (Ibidi; 200 μL/well) and incubated at 37°C for 6–8 h (depending on species). Germ-tubes formed in the wells were washed with phosphate-buffered saline (PBS, pH 7.4) twice and fixed with 2.5% *p*-formaldehyde (PFA; at room temperature for 1 h and then at 4°C overnight). Fixed germ-tubes were quenched with 0.1 M NH_4_Cl (thrice) and washed twice with PBS. Dormant spores were also fixed with PFA.

Fixed spores or germ-tubes were then used for Calcofluor White (CFW; Sigma) staining. Briefly, CFW (5 μg/mL in PBS; 200 μL/well) was added to the fungal samples (spores/germ-tubes) and incubated at room temperature for 10 min. Supernatant was discarded; fungal samples were washed with PBS and observed under confocal microscope (LSM 700, Zeiss). For lectin labeling, fluorescein isothiocyanate (FITC) conjugated Concanavalin A (ConA) or Wheat-Germ Agglutinin (WGA) (both from Sigma) at 5 μg/mL in PBS was added to fixed spores (1x10^5^/mL) or germ-tubes (50 μL for spores or 200 μL/well for germ-tubes), incubated at room temperature for 1 h. Then, the solutions were removed, and excess of ConA/WGA-FITC was removed by repeated washing with PBS, followed by subjecting the stained/lectin-labeled fungal samples to confocal microscopy.

For immunolabeling, fixed spores or germ-tubes in Ibidi plate wells were blocked with PBS-1% bovine serum albumin (BSA) at room temperature for 1 h. Mouse monoclonal anti-β-1,3-glucan antibodies (5 μg/mL in PBS) [27], mouse serum with polyclonal anti-α-1,3-glucan antibodies (1:100, in PBS) [28], and rat monoclonal EB-A2 antibodies [29] were added to fixed spores/germ-tubes for β-1,3-glucan, α-1,3-glucan, and galactomannan labeling, respectively, and incubated at room temperature for 1 h. Following, the fungal samples were washed (PBS, 3X), added with secondary antibodies conjugated with fluorochrome [mouse IgG conjugated with TRITC for β-1,3-glucan and α-1,3-glucan, and rat IgG-FITC for galactomannan (all from Sigma), incubated at room temperature for 1 h, washed thrice with PBS and observed under confocal microscopy.

### Cell wall (CW) analysis

Isolates were grown in a shaking incubator (150 rpm, 30°C, 24 h) upon inoculating 1x10^7^ spores in 30 mL RPMI-MOPS-2% glucose. The mycelia formed were collected by filtration, washed with sterile water to remove any medium, and broken by agitation with 1 mm glass beads at 6 m/sec for 60 sec, thrice, in a FastPrep (MP Biomedicals). From this lysate, cell wall fractions were collected by centrifugation at 3000 rpm for 10 min, and washed twice with water. The collected cell wall fractions were subjected to Tris (50 mM) – EDTA (50 mM) – SDS (2%)–β-mercaptoethanol (40 mM) (pH 7.4) treatment in a boiling water bath to remove membrane fractions and entangled proteins from the cell wall, to separate cell wall polysaccharides. Obtained cell wall polysaccharides were subjected to alkali fractionation by incubating at 70°C in 1 M NaOH containing 0.5 M NaBH_4_ for 1 h, twice. After each incubation, the supernatants collected after centrifugation were pooled, dialyzed against water, and freeze-dried [alkali-soluble (AS) fraction]. The pellets obtained after centrifugation were washed with water until neutrality and freeze-dried to obtain alkali-insoluble (AI) fraction.

AI/AS fractions were subjected to gas chromatography after derivatization, to determine their monosaccharide composition, following the protocol we described earlier [30]. After hydrolysis (with 4 N trifluoroacetic acid/8 N hydrochloric acid, at 100°C for 4 h) of the AI/AS fractions, the resultant monosaccharides were reduced and per-acetylated. Hexose composition in these derivatized samples was estimated by gas chromatography using Perichrom PR2100 Instrument (Perichrom) equipped with a flame ionization detector (FID) and a fused silica capillary column (30 m ×0.32 mm id) filled with BP1. The monosaccharide compositions (expressed in percent) in the AI or AS fractions were then calculated from the peak areas corresponding to different monosaccharides with respect to meso-inositol of known concentration (internal standard). A standard monosaccharide mixture containing the internal standard, subjected to acid hydrolysis, reduction, and derivatization was also passed to a gas chromatography column to identify different monosaccharides.

To characterize glucans in the AI fraction, in a total volume of 100 μL, AI fraction (10 μL; stock suspension, 10 mg/mL) was added with endo-β-1,3-glucanase (LamA), endo-β-1,6-glucanase [31] or amylase (Sigma) (2 μg each) in acetate buffer (pH 5.5) and incubated at 37°C for 60 h. The enzyme digests were then subjected to high-performance anion-exchange chromatography (DIONEX) [31]. The positive controls were LamA or endo-β-1,6-glucanase digested AI fractions of *A. fumigatus* and *S. cerevisiae* for β-1,3-glucan and β-1,6-glucan [31], respectively, whereas glycogen (Sigma) was the positive control for amylase digestion.

### Interaction with immune cells

THP-1 (human leukemia monocytic) cells from the frozen stock (3x10^6^/mL) were brought to 37°C, added with 9 mL of RPMI, mixed gently, and centrifuged at 1500 rpm for 5 min. The cell pellet was resuspended in 10 mL RPMI supplemented with 20% heat-inactivated foetal-bovine serum (HI-FBS), seeded into 75 cm^3^ culture flask, and incubated at 37°C with 5% CO_2_. After 3-days, cells were collected by centrifugation, counted, suspended in RPMI 1640 supplemented with 10% HI-FBS, and seeded into new flasks (5x10^5^-1x10^6^ cells per flask). After 4–5 passages, THP-1 cells collected were used for interaction study.

Our study involving human subjects adheres to the Declaration of Helsinki. Anonymized whole blood samples from healthy donors were obtained from the Etablissement Français du Sang (Paris, France) with written informed consent as per the guidelines provided by the Institutional Ethics Committee, Institut Pasteur (convention 12/EFS/023). Collected blood samples were used for the isolation of peripheral blood mononuclear cells (PBMC), and not for any clinical studies. PBMC was isolated from whole blood samples from healthy donors by Ficoll density-gradient separation (Eurobio, France) following the protocol described earlier [32]. PBMC or THP-1 cells (2x10^6^/mL) in RPMI supplemented with normal human serum (NHS; 10%) were seeded into a 96-well culture plate (100 μL/well). Metabolically active spores of *R. arrhizus*, *M. circinelloides* or *L. corymbifera* suspended in RPMI (1x10^6^/mL) supplemented with NHS (10%) were added to the wells containing PBMC or THP-1 cells (100 μL suspension per well) and incubated at 37°C with 5% CO_2_ for 24 h. THP-1 cells were also stimulated with cell wall fractions (AI and AS; 10 μg/well). Following, the culture supernatants were collected and stored at −20°C until further analysis. Different cytokines in the culture supernatants were estimated with sandwich ELISA using kits (DuoSet human cytokine detection kits; R&D Systems). THP-1 cells were also stimulated with Mucorales spores in RPMI medium devoid of NHS.

### Statistical analysis

Statistical analysis was performed using GraphPad Prism by one-way ANOVA with Dunnett’s post-test.

## Results

### Antifungal susceptibility and microscopic analysis of the spores

**Figure 1.**
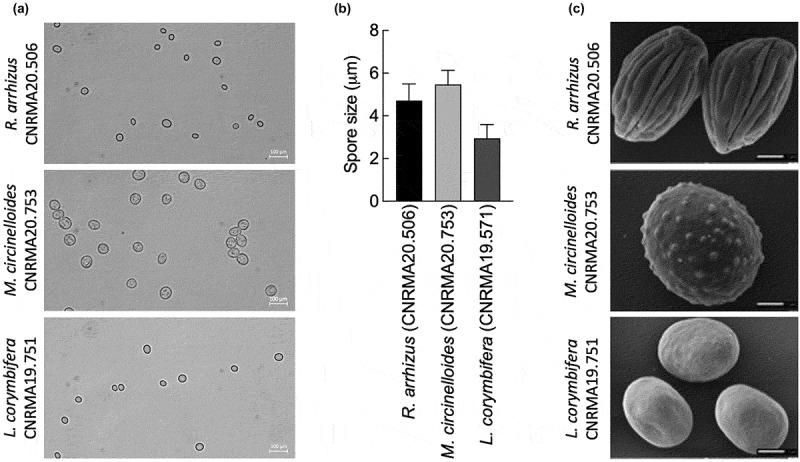
Morphological analysis of Mucorales spores. (a) Bright-field microscopy, (b) spore sizes, and (c) scanning electron microscopic (SEM) images of the three pathogenic species of Mucorales (scale bars, 1 μm). For each species, at least 100 spores were counted to determine their sizes.

Antifungal drug susceptibility of the three Mucorales species is presented in Table 1. All three species of Mucorales had the lowest MIC values for amphotericin B. Bright-field microscopy indicated that the spores of *M. circinelloides* were comparatively bigger and heterogeneous (5.46 ± 1.73 μm) than those of *L. corymbifera* (2.94 ± 0.64 μm), and *R. arrhizus* spores were intermediary in size (4.72 ± 0.78 μm) among the three species of Mucorales analyzed (Figure 1(a,b)). Scanning electron microscopic (SEM) images (Figure 1(c)) showed ridged spore surfaces for *R. arrhizus*, spikes for *M. circinelloides* spores, and smoother surfaces for *L. corymbifera* spores. These SEM observations for *R. arrhizus* and *M. circinelloides* spores were in agreement with the earlier reports [33,34].

### Spore surface-exposed polysaccharides

**Figure 2.**
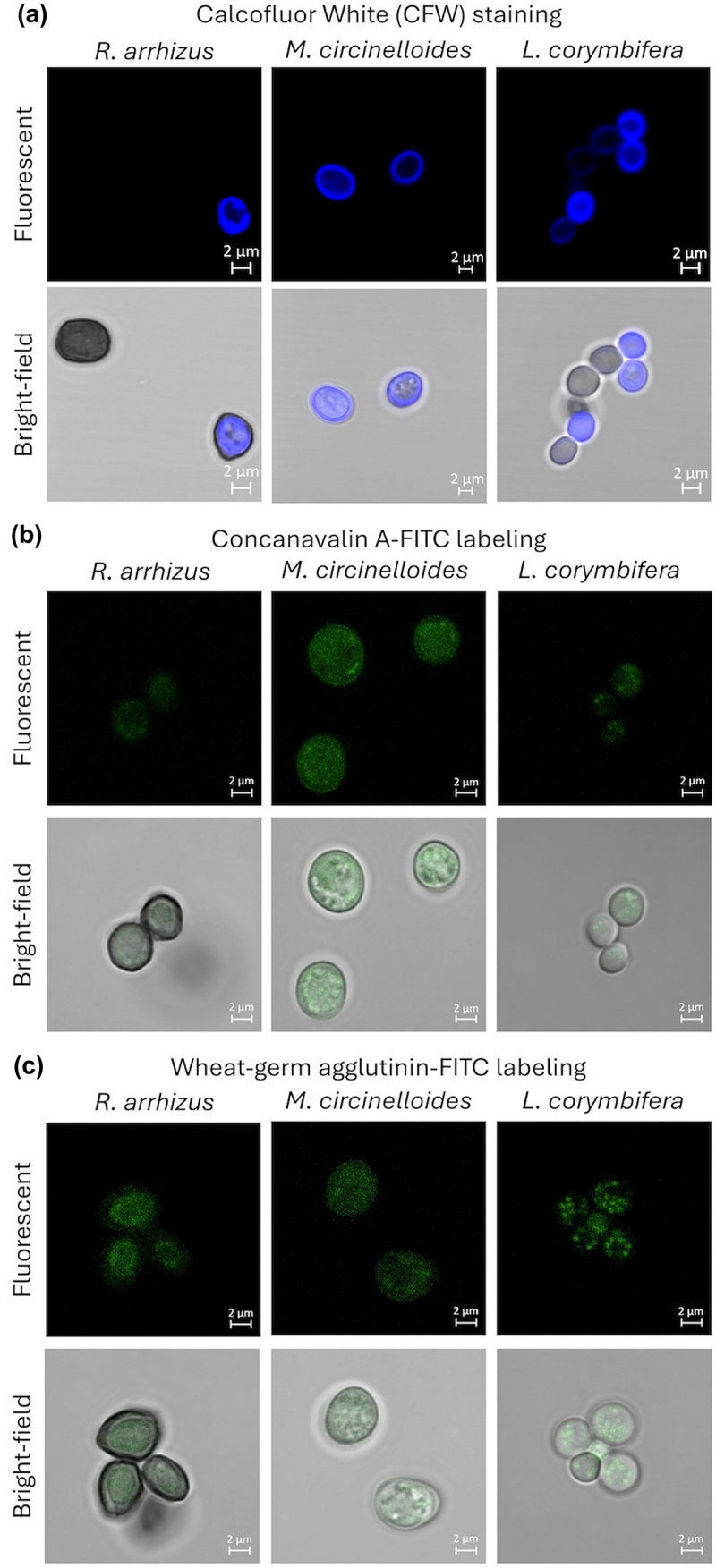
Confocal microscopic images of indicated Mucorales species spores. (a) staining with Calcofluor White (CFW; for cell wall chitin) and lectin labeling [(b) concanavalin-A (ConA) or (c) wheat-germ agglutinin (WGA), for surface-exposed mannans and chitin, respectively]; ConA and WGA are FITC-conjugated.

Mucorales spores were stained or labeled for surface-exposed cell wall polysaccharides [12]. With CFW that stains cell wall chitin, there was labeling of spores with variable intensities (Figure 2(a)). With WGA-FITC or ConA-FITC, which labels surface-exposed *N-*acetylglucosamine (building block of chitin) and mannose (monomeric unit of mannan) residues, respectively, there was punctate labeling of spores from all the three species of Mucorales (Figure 2(b,c)). On the other hand, spores from all the three Mucorales species showed negative immunolabeling with 5H5 (monoclonal mouse anti-β-1,3-glucan antibody) or mouse serum with polyclonal anti-α-1,3-glucan antibodies (raised against synthetic α-1,3-pentasaccharide) [27,28]. Together, these data suggest that β-1,3-glucan and α-1,3-glucan are not exposed on the surfaces of *R. arrhizus*, *M. circinelloides* and *L. corymbifera* spores, while mannan and chitin are present on their spore surfaces but with heterogeneous distribution.

### Polysaccharide exposure during germination

**Figure 3.**
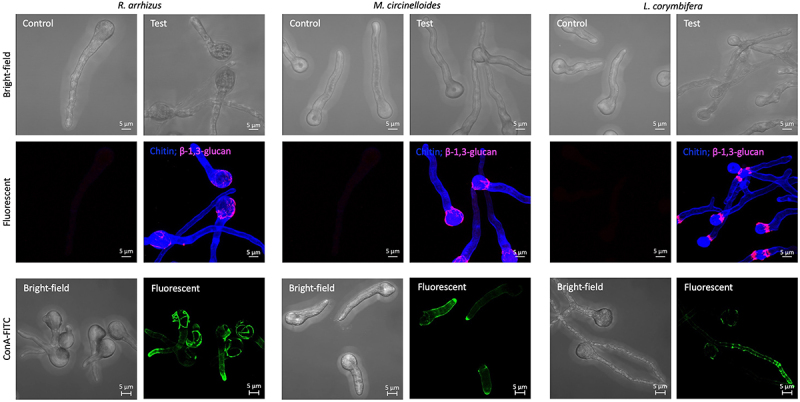
Confocal microscopy images of indicated Mucorales species germ-tubes: staining with Calcofluor White (for staining cell wall chitin), immunolabeling for germ-tube surface-exposed β-1,3-glucan with mouse monoclonal antibody 5H5 and secondary mouse IgG-TRITC (red) (chitin and β-1,3-glucan labeling are merged); lectin ConA labeling (conjugated with FITC) for germ-tube surface-exposed mannans (in green).

Germ-tubes were stained/labeled for cell wall and surface-exposed polysaccharides (Figure 3). CFW staining showed uniform distribution of chitin, whereas WGA-FITC labeling was negative, suggesting that the germ-tubes do not expose chitin on their surfaces. Labeling with ConA-FITC was positive for all the three species of Mucorales. Labeling was observed mostly on the germ-tubes suggesting that the germ-tube surfaces are composed of mannan. Immunolabeling with 5H5 antibodies for β-1,3-glucan showed positive labeling only on the spore heads for *R. arrhizus* and *M. circinelloides*, and at the junction of spore head and germ tube for *L. corymbifera*. The β-1,3-glucan labeling pattern observed for *R. arrhizus* was comparable to that reported earlier [18]. Immunolabeling of germ-tubes with polyclonal anti-α-1,3-glucan antibodies or EB-A2 (rat monoclonal antibodies raised against galactomannan from *A. fumigatus*) was negative. This suggested the absence of surface-exposed α-1,3-glucan and galactomannan, or these polysaccharides are structurally distinct compared to those of *A. fumigatus*.

### Cell wall analysis

**Figure 4.**
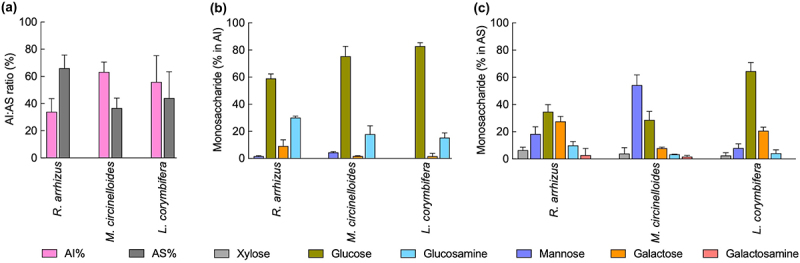
Mycelial cell wall analysis: (a) ratio of alkali insoluble (AI) and soluble (AS) fractions, (b and c) monosaccharide composition in the AI and AS fractions, respectively; three biological replications were performed for each isolate.

Fungal cell wall polysaccharides generally could be categorized as fibrillar and amorphous components, and they could be extracted as alkali-insoluble (AI) and alkali-soluble (AS) fractions, respectively. Although not statistically significant, the AS fraction was comparatively higher in content than that of AI fraction for *R. arrhizus*, lower for *M. circinelloides* and similar for *L. corymbifera* (Figure 4(a)). We then determined the monosaccharide composition in the two cell wall fractions. Regardless of the species, glucose was the major monosaccharide present in the AI fraction followed by glucosamine (Figure 4(b)), while mannose and galactose showed species distribution: in *R. arrhizus*, galactose was higher than mannose, which was the opposite in *M. circinelloides*, and in *L. corymbifera* only galactose was detectable and not mannose. On the other hand, AS fraction showed species-specific monosaccharide composition. Glucose was the major monosaccharide in *R. arrhizus*, followed by galactose and mannose; in *M. circinelloides*, the major monosaccharide was mannose, followed by glucose and galactose; in *L. corymbifera*, glucose was the major monosaccharide, followed by galactose and mannose. Glucosamine, galactosamine, and xylose were the other monosaccharides identified in the AS fractions; nonetheless, galactosamine was not detectable in the AS fraction of *L. corymbirera* (Figure 4(c)). These data suggest that the fibrillar cell wall components of the three Mucorales species are comparable, but not that of their amorphous components.

**Figure 5.**
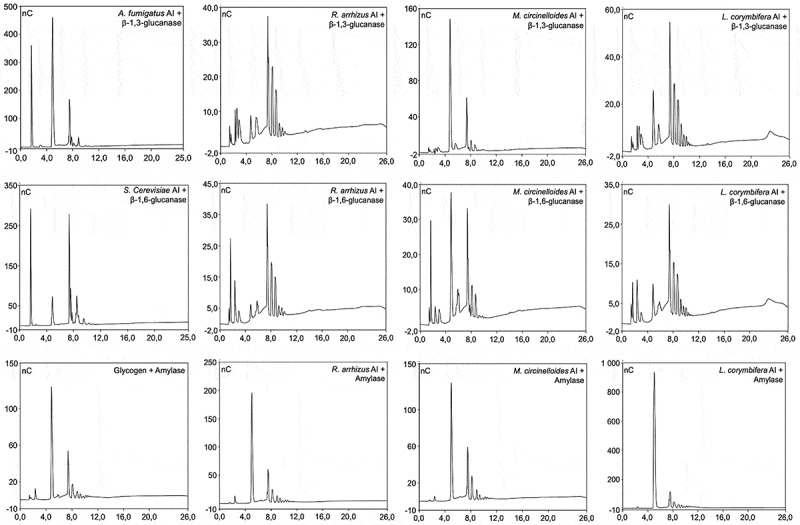
Digestion of mycelial alkali-insoluble (AI) fractions from *R. arrhizus*, *M. circinelloides* and *L. corymbifera* with β-1,3-glucanase, β-1,6-glucanase, and amylase. *A. fumigatus* AI fraction digested with β-1,3-glucanase, *S. cerevisiae* AI fraction digested with β-1,6-glucanase, and glycogen digested with amylase were used for the controls for β-1,3-glucan, β-1,6-glucan and glycogen digestion patterns.

As glucose was the major monosaccharide present in the AI fractions of the three Mucorales species, which represent β-1,3-glucan in other filamentous fungi [35], we sorted to determine the nature of glucan polymer in the AI fractions of the Mucorales. We subjected the extracted AI fractions to β-1,3-glucanase and β-1,6-glucanase digestion followed by colorimetry (phenol-H_2_SO_4_) [36] and high performance anion-exchange chromatography (DIONEX) of the enzyme digests. As control, AI fractions from *A. fumigatus* and *Saccharomyces cerevisiae* were used for β-1,3-glucanase and β-1,6-glucanase digestion, respectively. *A. fumigatus* cell wall is composed mainly of branched β-1,3-glucan and that of *S. cerevisiae* is composed of both branched β-1,3-glucan and β-1,6-glucan [31]. AI fractions from all the three species of Mucorales showed β-1,3-glucanase digestible β-1,3-glucan (Figure 5, upper panel). However, β-1,3-glucanase could digest ~ 23, 42 and 28% of glucan in the AI fractions of *R. arrhizus*, *M. circinelloides*, and *L. corymbifera*, respectively. This led us to ask what are the other possible structures of β-glucans in these Mucorales. As β-1,6-glucan is the other possible glucan polymer in the fungal cell wall, we subjected AI fraction of these Mucorales to β-1,6-glucanase digestion (Figure 5, middle panel). β-1,6-Glucanase digestion released ~ 24, 18 and 14% of the glucan polymer from the AI fractions of *R. arrhizus*, *M. circinelloides*, and *L. corymbifera*, respectively. As β-1,3-glucanase and β-1,6-glucanase digests were not accounting for 100% of the AI fraction, we further checked the possibility of storage polysaccharide, glycogen, as the cell wall constituent. Therefore, we digested AI fractions of *R. arrhizus*, *M. circinelloides*, and *L. corymbifera* with amylase (Figure 5, lower panel) that released about 44, 33, and 55% of the glucan polymer, respectively. This suggested that β-1,3-glucan, β-1,6-glucan, and amylase digestible glucan constitute major glycan polymers in the cell walls of the three Mucorales species analyzed. We subjected the AS fraction from these three species of Mucorales for β-1,3-glucanase, β-1,6-glucanase, and amylase treatment as well. They also showed these enzymes digestible glucans, but at lower levels compared to their respective AI fractions. Together, this cell wall analysis indicated that the Mucorales have species-specific cell wall composition.

### Immunostimulatory potentials

**Figure 6.**
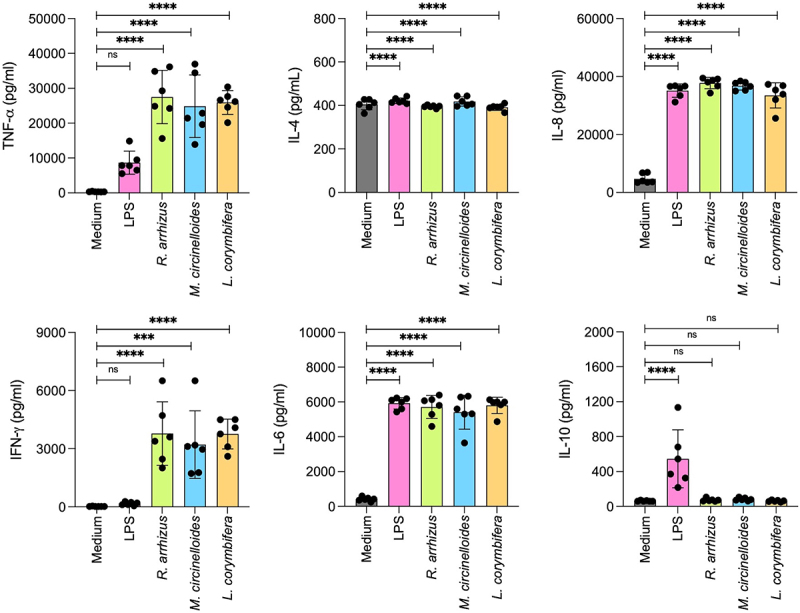
Cytokine secretion by peripheral blood mononuclear cells (PBMCs) isolated from healthy human blood samples (number of donors = 6) stimulated with spores of *R. arrhizus, M. circenelloides,* and *L. corymbifera* isolates; *p < 0.05, **p < 0.005, ***p < 0.001, and ****p < 0.0001.

**Figure 7.**
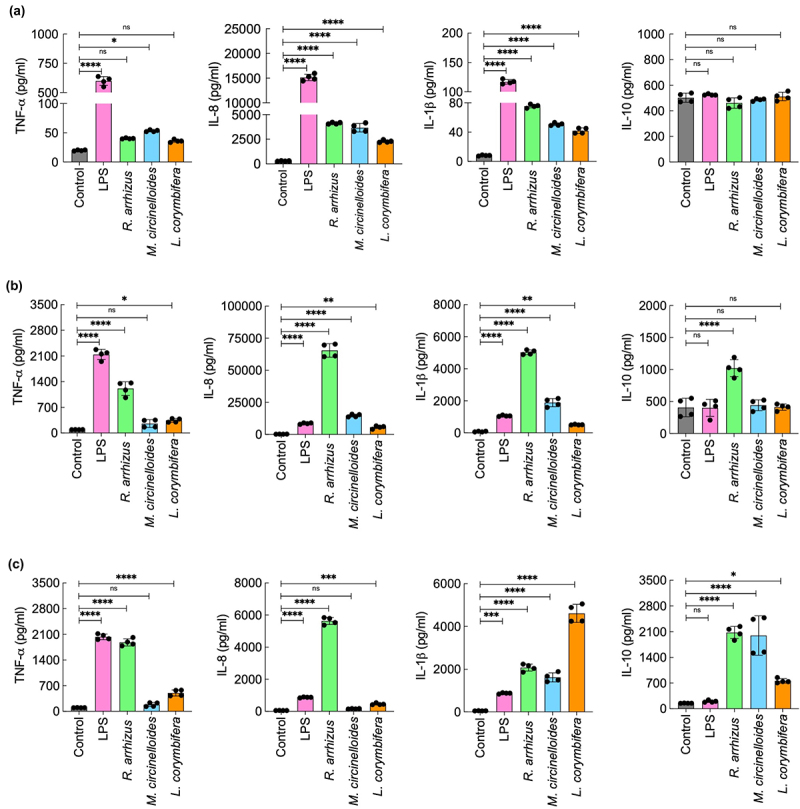
Cytokine secretion by THP-1 cells stimulated with (a) spores of AI and AS fractions [(b) and (c), respectively] extracted from *R. arrhizus, M. circinelloides,* and *L. corymbifera* isolates. Four biological replicates were performed; *p < 0.05, **p < 0.005, ***p < 0.001 and ****p < 0.0001.

Further, we investigated immunomodulatory capacity of the three Mucorales spores. PBMCs stimulated with spores resulted in the secretion of pro-inflammatory cytokines (TNF-α, IFN-γ, IL-6), chemokine (IL-8), but not anti-inflammatory cytokine analyzed (IL-10) or the Th2 cytokine IL4 (Figure 6). Similarly, THP-1 cells stimulated with Mucorales spores from the three species also secreted pro-inflammatory cytokines (TNF-α and IL-1β) and IL-8 (Figure 7(a)), but not IL-10. Interestingly, in the medium devoid of serum, THP-1 cells failed to secrete any cytokines/chemokines upon stimulation with spore of all the three species of Mucorales, suggesting that the serum factors could be necessary for the immunomodulation by the Mucorales.

Further, we analyzed immunomodulatory capacities of the alkali-insoluble (AI) and soluble (AS) fractions of the Mucorales cell walls. As both PBMCs and THP-1 cells when stimulated with Mucorales spores resulted in comparable cytokine profiles (i.e. significant stimulation of the pro-inflammatory, but not anti-inflammatory, cytokines), immunomodulations by AI and AS fractions were studied using THP-1 cells (Figure 7(b,c)). AI fractions from all the three species of Mucorales increased the release of pro-inflammatory cytokines (TNF-α, IL-1β) and IL-8 from THP-1 cells; AI fraction from *R. arrhizus* also increased the release of IL-10, whereas the AS fractions from three Mucorales species showed distinct responses: AS fraction from *R. arrhizus* and *L. corymbifera* stimulated the release of both pro- and anti-inflammatory cytokines as well as IL-8, while that of *M. circinelloides* stimulated the secretion of IL-1β and IL-10, but not TNF-α and IL-8. Together, all these data indicated that the cell wall components of the three Mucorales species analyzed are highly pro-inflammatory.

## Discussion

Here, we focused on the most frequent pathogenic species of Mucorales, analyzed the exposure of different cell wall components on their spores as well as germ-tube surfaces, and determined the composition of their cell walls and immunostimulatory potentials of their intact spores as well as their extracted cell wall fractions. Our study indicated species-dependent differences in their spore sizes, surface exposure of different polysaccharides, and cell wall compositions. However, and regardless of the species, the three Mucorales species (both spores and their cell wall fractions) were highly pro-inflammatory in their immunomodulatory capacity.

Earlier, using reference/clinical isolates it was shown by others that the spores of *R. arrhizus* and *L. corymbifera* induce pro-inflammatory immune response when interacting with human mononuclear phagocytes [37,38]. This is in agreement with our observation with the clinical isolates we studied. However, in this study, our focus was on the fungal cell wall, which protects the fungal cell and is the first component to interact with the host immune system. In general, fungal cell wall is composed mainly of polysaccharides that can be pan-fungal or species-specific [39]. Fungal cell wall polysaccharides can be pathogen-associated molecular patterns (PAMPs). When recognized by the host pattern-recognition receptors (PRRs), these polysaccharides modulate host immune responses. Importantly, immunomodulation is PAMP specific; for example, in *Aspergillus fumigatus*, another airborne fungal pathogen, its major fibrillar cell wall polysaccharide, β-1,3-glucan, stimulates a protective Th1 response [40], and its amorphous cell wall polysaccharide α-1,3-glucan polarizes regulatory T cells [41], thus playing a crucial role in the immunomodulation, thereby maintaining an immune balance. Therefore, we investigated the exposure of different cell wall polysaccharides on the spores (infective propagules) as well as on germinating spores (morphotype when infection is established) of *R. arrhizus*, *M. circinelloides,* and *L. corymbifera*. Staining of their cell wall chitin with chitin binding dye CFW was homogeneous for *M. circinelloides*, while *R. arrhizus* and *L. corymbifera* showed heterogeneous staining (~50% spores were CFW-positive). However, GC analyses identified the presence of chitin in the cell walls of all the three species of Mucorales. Thus, differential staining of the spores could be attributed to the heterogenous surface organization of cell wall chitin. Immunolabeling for β-1,3-glucan and α-1,3-glucan was negative, suggesting that these two polysaccharides are not exposed on their spore surfaces. Lectin labeling showed punctate exposure of ConA- or WGA-positive polysaccharides; these lectins recognize mannose and N-acetylglucosamine moieties, the building blocks of mannan and chitin, respectively, suggesting the exposure of mannan and chitin on the surfaces of spores.

On the other hand, the germ-tubes of the three Mucorales species showed uniform CFW staining, but negative labeling for WGA, suggesting deeper localization of chitin in the germ-tube cell wall. ConA labeling for cell wall mannan showed labeling of the germ-tube, but not to their spore heads. While immunolabeling for cell wall β-1,3-glucan was positive on spore head for *R. arrhizus*, at the spore head-germ tube junction for *M. circinelloides* and *L. corymbifera*. This was similar to the exposure of β-1,3-glucan in the bud scars of *Candida albicans* [42] that may serve protective function or facilitate targeted phagocytic attack. A recent study reported exposure of β-1,3-glucan on the surface of swollen and germinating spores of *L. corymbifera* [38]. However, the pattern was more uniform in contrary to localized exposure that we observed. This discrepancy could be due to the monoclonal antibodies (mAbs) used in the two studies. We used mouse mAb raised against a synthetic β-1,3-nonamer; the smallest epitopes recognized by this mAb are linear β-1,3-trimer and β-1,6-branched β-1,3-octamer [27]. The mouse mAb used in the previous study [38] was generated using β-glucan extracted from *C. albicans* cell wall, a mixture of β-1,3-glucan and β-1,6-glucan. We also performed immunolabeling of the Mucorales germ-tube with polyclonal anti-α-1,3-glucan antibodies and monoclonal anti-galactomannan antibodies (EB-A2) specific for *A. fumigatus*. Immunolabeling with both was negative, suggesting that (i) either Mucorales species we analyzed are devoid of surface-exposed α-1,3-glucan or this polysaccharide is absent in their cell walls, and (ii) galactomannans of Mucorales are structurally distinct from the galactomannan that is found in the cell wall of *A. fumigatus*.

Fungal cell wall is composed of fibrillar and amorphous polysaccharides, which can be extracted as alkali-insoluble (AI) and soluble (AS) fractions, respectively. Polysaccharides in the AS fraction are species-specific, whereas AI fraction contains generally pan-fungal polysaccharides such as β-1,3-glucan and chitin [43]. We performed monosaccharide composition analysis of the cell wall of Mucorales mycelia, the morphotype that establishes infection. Although not significant, in *M. circinelloides* and *L. corymbifera*, AI fraction was in a higher proportion than AS fraction. *R. arrhizus* showed lower AI content compared to AS fraction. The three Mucorales species showed glucose as the major monosaccharide in their cell wall AI fractions, followed by glucosamine. Their AS fractions showed species-dependent composition: glucose was the major monosaccharide present in *R. arrhizus* and *L. corymbifera*, while in *M. circinelloides*, mannose was the major monosaccharide followed by glucose. *R. arrhizus* and *L. corymbifera* showed differences in the proportions of mannose. The presence of uncommon monosaccharides such as fucose, galactose, and glucuronic acid has been reported in the cell walls of a reference strain and a clinical isolate of *L. corymbifera*, which we did not identify. This is possibly due to the differences in the protocols and techniques used in the two studies [38]. On the other hand, for the first time we report the presence of amylase digestible polysaccharide as a constituent in the cell walls of *R. arrhizus*, *M. circinelloides,* and *L. corymbifera*. Recent studies have shown the presence of glucan–glycogen complex as a constituent in the cell walls of several *Candida* and *Sporothrix* species [44,45]. β-1,3-Glucan forms triple helix, but glycogen is a highly branched polymer. Its integration in the cell wall may, in-part, explain why Mucorales show high level of resistant to multiple antifungal agents [46], as glucan–glycogen complex may decrease cell wall permeability. Interestingly, glucan–glycogen complex was found in the cell walls of *C. auris, C. dublinensis*, *C. haemulonii*, but not of *C. albicans* and *C. glabrata*. Although *C. dublinensis* is sensitive, *C. auris* and *C. haemulonii* exhibit resistance to antifungals [47,48], supporting that cell wall glucan–glycogen complex has a role in the antifungal susceptibility. This hypothesis needs to be validated experimentally.

Mucormycosis is an angioinvasive infection, and its diagnosis is challenging. Detection of circulating antigens, specifically fungal cell wall polysaccharides, is routinely used for the diagnosis of fungal infection. However, there are no circulating antigen-based detections available for mucormycosis, like galactomannan detection for invasive aspergillosis [49]. It agrees with our data that Mucorales were negative for immunolabeling with EB-A2, a galactomannan-specific monoclonal antibody. This suggests that Mucorales lacks galactomannan or their galactomannan structure is different from that of *A. fumigatus*. On the other hand, the detection of β-1,3-glucan, the other circulating molecular biomarker for diagnosis of invasive fungal infections, is not sufficiently powered/cannot be detected during the infection by Mucorales [50]. This also agrees with our immunolabeling study for β-1,3-glucan, as its exposure was restricted to the spore heads and spore head–germ tube junction. Moreover, specific enzymatic digestion indicates that the glucan in the AI fraction is not only β-1,3-glucan, but also composed of β-1,6-glucan and amylase digestible glucan. As the extraction of AI fraction involves harsh chemical treatments, amylase digestible glucan identified in the Mucorales cell wall could not be a contaminant, but a true component of the AI fraction. Moreover, chromatography profiles of the β-1,3-glucanase and β-1,6-glucanase digested AI fractions of Mucorales were distinct from those of *A. fumigatus* and *S. cerevisiae*. This suggests that the structure of glucans in the Mucorales cell wall is different compared to *A. fumigatus* and *S. cerevisiae*, which needs to be evaluated further by gas chromatography–mass spectrometry analyses.

We examined immunomodulatory potentials of spores as well as the cell wall fractions of the three Mucorales species. The relevance of studying the immunostimulatory potential of AI and AS fractions lies in understanding their specific immunomodulatory potential. Moreover, the capacity of cell wall components to elicit cellular and humoral immune responses has been exploited in the serodiagnosis of mycoses, and the identification of fungal species responsible for infection has been explored in the development of fungal vaccines [51]. We used both primary immune cells (PBMCs) and leukemic monocytes (THP-1) cells in our study. All the three species of Mucorales resulted in the stimulation of mainly pro-inflammatory cytokines from both these immune cells. Nonetheless, PBMCs and THP-1 cells stimulated with Mucorales spores secreted different levels of cytokines compared to lipopolysaccharide (LPS; positive control). This is in agreement that these cells differ in cytokine secretion upon LPS stimulation [52], suggesting a need to select the immune cells for *in vitro* study. However, among the spores of three Mucorales there was no difference in the trend of cytokine secreted by PBMCs and THP-1 cells. Moreover, though not significant, AI and AS fraction showed batch-to-batch variation within three replicates of a Mucorales species. Unlike PBMCs that exhibit donor-to-donor variabilities, THP-1 cells provide consistent and reproducible model system. We aimed at keeping one of the components constant to account for the variability of the other interactome (AI/AS fraction). Therefore, immunostimulatory potentials of cell wall fractions of these Mucorales species were studied using THP-1 cells.

Stimulation of THP-1 cells with specific cell wall fractions also led to the release of mainly pro-inflammatory cytokines. Interestingly, in the culture medium devoid of serum, the Mucorales spores failed to stimulate any cytokine secretion from THP-1 cells, suggesting that humoral immune components play a crucial role in their recognition. Indeed, a very recent study demonstrated that *R. arrhizus*, *M. circinelloides,* and *L. corymbifera* interact with complement components C1q, C3, and terminal complement complex (C5b-C9) [53]. However, their interaction varies with the species, while complement deficiency is a risk factor for mucormycosis in a murine model [53]. A recent study reported that two isolates of *L. corymbifera* were highly pro-inflammatory [38]. This leads to the speculation that the hyperinflammation due to Mucorales species (specifically due to their constituent cell wall polysaccharides) may favor the progression of mucormycosis, but this hypothesis needs to be validated. In a murine model of intranasal infection, immunopathogenesis was shown to be species-specific, and affected by immunosuppression or underlying risk [54]. Immunosuppressed mice survived longer when infected with *R. arrhizus* compared to *L. corymbifera*, while diabetic mice survived longer when infected with *L. corymbifera* compared to those mice infected with *R. arrhizus* [54].

A limitation of our study is the analysis of one clinical isolate for each of the three Mucorales species. Although not presented, we analyzed two clinical isolates of *L. corymbifera* collected from two different sites of infection (lung and sinus). They did not show differences in their drug susceptibilities, morphology, staining or labeling patterns, cell wall composition, and immunostimulatory properties. However, our preliminary study on additional clinical isolates from other two species of Mucorales, collected from different sites of infection, showed different antifungal susceptibility. Although not statistically significant, these clinical isolates displayed minor differences in their cell wall compositions. Therefore, future studies need attention to consider isolate specific/dependent drug susceptibility and possible heterogeneity in their immunostimulatory capacity.

In conclusion, we demonstrate that the pathogenic species of Mucorales exhibit differential exposure of their cell wall components during germination and species-specific monosaccharide composition. Nonetheless, all the three Mucorales species result mainly in a pro-inflammatory immune response. Moreover, their recognition could be preferentially soluble PRRs rather than immune cell surface-expressed PRRs. Our study providing this important information should lay a base for in-depth analysis of cell wall glycobiology and immunobiology of the three most frequent pathogenic species of Mucorales.

## Data Availability

Clinical isolates of Mucorales used in this study could be obtained from the National Reference Centre for Invasive Mycoses and Antifungals (CNRMA), Institut Pasteur Paris. Data related to the bar-graphs (Figure 1b, 4, 6, and 7) can be accessed through https://doi.org/10.6084/m9.figshare.28095443.
